# Effectiveness of Virtual Reality–Based Cognitive Control Training Game for Children With Attention-Deficit/Hyperactivity Disorder Symptoms: Preliminary Effectiveness Study

**DOI:** 10.2196/66617

**Published:** 2025-09-19

**Authors:** Hyunjoo Song, Yunhye Oh, JongIn Choi, Seong-Yong Ohm

**Affiliations:** 1 Division of Psychology and Cognitive Science, Seoul Women's University, Hwarang Ro 621, Nowon Goo, Seoul, 01797, Republic of Korea, 82 029705888; 2 Department of Psychiatry, Hallym University Sacred Heart Hospital, Republic of Korea; 3 Department of Digital Media Design and Application, Seoul Women's University, Seoul, Republic of Korea; 4 Department of Software Convergence, Seoul Women's University, Seoul, Republic of Korea

**Keywords:** cognitive control, virtual reality, serious game, attention-deficit/hyperactivity disorder, ADHD, training effectiveness

## Abstract

**Background:**

Recent advancements in digital technologies hold promise for psychological interventions. Virtual reality (VR) has emerged as a particularly innovative tool, and its application expanded during the COVID-19 pandemic period. A recent study combining material and psychological rewards within a VR platform showed that this approach effectively improves attention-deficit behaviors in children with attention-deficit/hyperactivity disorder (ADHD), enhancing their inhibitory control abilities.

**Objective:**

This study aimed to evaluate the effectiveness of a newly developed VR-based cognitive control training game for children with ADHD symptoms. Specifically, it examined the sustainability of the training effects through a 3-month follow-up assessment. In addition, the study analyzed training response patterns and influential factors using a clustering method.

**Method:**

A total of 29 children and adolescents (21 males and 8 females) aged 10-14 years participated in the study, with a mean IQ of 94 (SD 16.53). For 20 consecutive days, participants self-administered the training on a daily basis using the VR app. The following assessments were administered face-to-face: the Korean Wechsler Intelligence Scale for Children, Fourth Edition; the Stroop test; the Color Trails test; and the Flanker test from the National Institutes of Health toolbox. In addition, the parent-completed Korean Child Behavior Checklist was used to identify behavioral problems in the children. Participants engaged in at least 20 minutes of daily training for 20 consecutive days, with assessments conducted at baseline, posttraining, and follow-up.

**Results:**

Repeated measures ANOVA revealed significant main effects in the Stroop Color-Word test (*F*_2,56_=4.97; *P*=.001; ηp^2^=0.151), Child Behavior Checklist (CBCL) Total Problems (*F*_2,56_=21.0; *P*<.001; ηp^2^=0.429), CBCL Attention Problems (*F*_2,56_=11.7; *P*<.001; ηp^2^=0.294), and CBCL ADHD (*F*_2,56_=3.46; *P*=.004; ηp^2^=0.110). K-means clustering identified 2 distinct clusters that did not differ significantly in IQ variables but showed significant differences in game-related behavioral variables, including mean correct response time (*t*_27_=−2.56; *P*=.02) and the correct response ratio (*t*_27_=2.60; *P*=.02).

**Conclusions:**

The findings indicate that the VR-based training effectively improved cognitive control on the Stroop test and ADHD-related symptoms as measured by the CBCL. However, no significant training effects were observed on other attentional measures, namely the Color Trails test and the Flanker test from the National Institutes of Health toolbox. This VR-based approach shows promise as a potential therapeutic intervention for children with ADHD symptoms.

## Introduction

Technological advancements have significantly transformed psychological interventions, particularly through digital therapeutics and personalized treatment strategies. The concept of “enhanced psychotherapy” has been introduced, which builds on traditional psychotherapy by integrating innovative treatment modules, approaches, or techniques [1]. A distinctive feature of this enhanced psychotherapy is the use of personalized treatment strategies, incorporating digital technologies, wearable devices, and computerized cognitive behavioral therapy. Recent advancements in digital technologies hold significant promise for psychological interventions. For instance, the integration of formally recognized treatment options, such as EndeavorRX (Akili Interactive; AKL-T01), which has received approval from the Food and Drug Administration, has enhanced both the feasibility and applicability of digital therapeutics in clinical practice [2]. It has been argued that current technologies could transform cognitive interventions, allowing for better targeting of individuals in need of specific treatments [3]. In addition, it has been emphasized that the ability to dynamically tailor treatments in real-time through data processing technologies marks the dawn of a new era in psychiatry [4].

Virtual reality (VR) has emerged as a particularly innovative tool [5]. It has revolutionized psychotherapy and psychological assessment [6,7], and its application significantly expanded during the COVID-19 pandemic, including in sensitive contexts [8-10]. VR interventions have been explored in the treatment of anxiety disorders, pain management, eating disorders, psychosis, autism, and addiction. Research suggests that VR-based interventions can be as effective as, or even more beneficial than, traditional treatment methods [11]. Several recent studies have highlighted the potential of VR in cognitive intervention. For instance, attention training using VR has been reported to be effective in patients with acquired brain injuries [12]. Research has demonstrated that VR technology provides significant benefits in mental health management for adolescents experiencing various challenges, including emotional, cognitive, and social difficulties [13,14].

Although treatments for attention-deficit/hyperactivity disorder (ADHD) have been rigorously documented [15], therapies focusing on enhancing cognitive control lack systematic validation regarding both efficacy and long-term treatment effects [16]. In addition, there is a notable shortfall in ecologically valid treatment outcomes. Basically, treatment benefits should translate into improvements in real-world functioning. Spooner and Pachana [17] stressed that neuropsychological assessment should correlate closely with daily functioning outcomes. However, a recent meta-analysis reflecting on 2 decades of research identified only 7 studies that included parental reporting on ADHD symptoms and behaviors in daily life [18].

Cognitive control is a foundational concept in modern cognitive neuroscience, encompassing decision-making, goal-directed behavior, response inhibition, and attention allocation [19]. Deficits in cognitive control have been closely linked to childhood and adolescent behavioral problems, including ADHD [20-22], autism spectrum disorder [23], conduct disorder, and various impulsive behaviors [24]. ADHD is notably associated with deficits in cognitive control, which can be identified through behavioral assessments and neuroimaging measures [25]. The prefrontal cortex, with its extensive interconnectivity with sensory, motor, and subcortical structures, serves as a hub for cognitive control [19,20,26].

One study illustrated VR’s potential in treating ADHD, showing results comparable to those achieved with methylphenidate treatment [14]. In addition, a recent study combining material and psychological rewards within a VR platform showed that this approach effectively improves attention-deficit behaviors in children with ADHD, enhancing their inhibitory control abilities [26]. Furthermore, research has demonstrated that older adults experiencing cognitive and functional decline benefit from VR game interventions, with particularly pronounced effects observed in self-adaptive VR interventions [27]. This highlights the importance of participant-centered approaches in optimizing both cognitive and motor outcomes while prioritizing the enhancement of user experiences. The recent review exploring VR and exercise simulator intervention in patients with ADHD emphasized the need for a comprehensive approach to interventions and advocated improving technological interventions to address the varied needs of individuals with ADHD [28,29].

**Figure 1.**
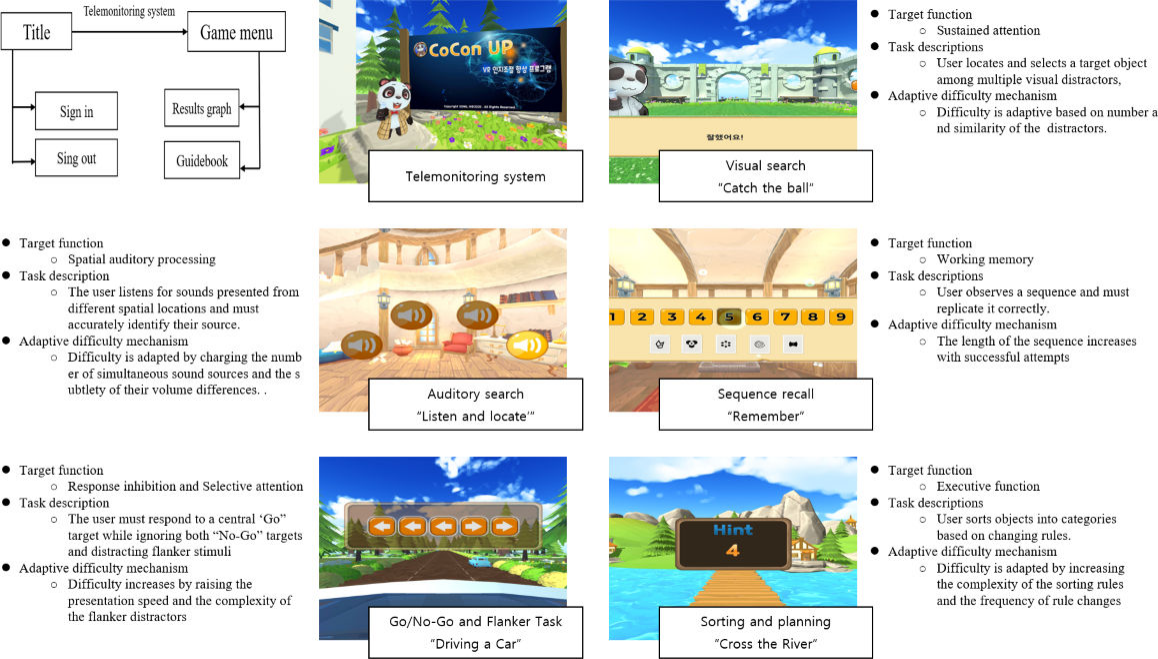
Key components of the game's architecture.

We hypothesized that the immersive experience of VR could offer an optimal training environment for children with ADHD symptoms, particularly those who struggle to maintain focus due to difficulties in filtering irrelevant stimuli. In addition to the immersive experience, the newly developed VR game encompasses 3 core aspects. First, the intervention is built on rigorous cognitive neuroscience principles. This VR game was designed based on carefully selected games from the cognitive control training app, CoCon (Huno [30]). CoCon, a previously validated 2D mobile app, has shown promising results in improving cognitive control in children, thus providing a strong foundation for this VR-based extension. Each game was designed based on a cognitive experimental paradigm relevant to cognitive control functions, such as visual and auditory search, working memory, response inhibition, and executive function [30]. Second, this VR game uses an adaptive difficulty algorithm, ensuring that participants benefit from the training regardless of their baseline attention capabilities. The game difficulty progressively increases as users advance, pausing when users reach their cognitive capacity limits. Task difficulty is regulated using algorithms derived from an adaptive staircase approach. The adaptive staircase algorithm, originally developed based on established psychophysical methods and now patented (10-2019-0125031), enables real-time adjustment of task difficulty to individual cognitive thresholds. Third, this VR game incorporates a remote monitoring system, encouraging consistent engagement in the training program. Research assistants track participants’ progress through server data and provide feedback via phone, engaging primary caregivers when necessary. This integrated approach of at-home training, combined with direct support, has been designed to optimize treatment outcomes. The overall structure and primary cognitive functions of each VR game are depicted in Figure 1. This figure shows the main modules of the game system, including the user interface, the core gameplay loop, the data management module, and their interconnections.

This study aims to examine the efficacy of the VR-based cognitive training through a structured 20-day intervention program, followed by a 3-month follow-up assessment to evaluate the durability of training effects. We hypothesize that the intervention will yield significant and sustained improvements in key domains of cognitive control, including attention regulation, inhibitory control, and executive functioning.

## Methods

### Participants

A total of 32 children aged 10-14 years participated in this study. This age range was selected to ensure participants had the developmental capacity to understand instructions, operate the VR equipment, and adhere to the training protocol. A total of 22 participants were recruited from the Department of Psychiatry at a general hospital in Seoul, where they were diagnosed with ADHD by a child psychiatrist. None had comorbidities. Notably, 18 participants were medicated with varying types and dosages, while 4 were medication-naive. An additional 10 participants were recruited from the community via social networks and community boards. Inclusion criteria for community participants were based on the mobile-based cognitive control assessment app, CoCon. To be included, candidates had to score below a T-score of 40 on at least one of 4 indices: sustained attention, working memory, cognitive control, and cognitive execution. A T-score of 40 on the CoCon app represents a mean score 50 (1 SD) below the mean. There were no significant differences between the clinical and community participants in the basic variables (Table S1 in Multimedia Appendix 1).

Sample size calculation was performed using G*Power version 3.1 software (Heinrich-Heine-Universität Düsseldorf [31]). Initially, the study was designed as a between-subjects repeated measures ANOVA, and a sample size of 86 was recommended, assuming a medium effect size (Cohen *f*=0.25), an α level of .05, and a statistical power of 0.80. However, due to uncontrollable circumstances, such as the COVID-19 pandemic, it was not feasible to recruit enough participants for the between-subjects design. Therefore, the study design was changed to a within-subjects repeated measures ANOVA. Based on this revised design, a sample size of 28 was recommended under the same parameters (Cohen *f*=0.25; *α*=.05; power=0.80), assuming a medium effect size for a within-subjects context. To account for an estimated dropout rate of 20%, the final target sample size was increased to 32 participants. Overall, 3 participants dropped out during training, resulting in a final count of 29 participants. In addition, 1 participant who reported red-green color blindness after the pretraining test had their Stroop test data replaced with mean values from corresponding variables for analysis. For the 29 participants who completed all study assessments, all of them also completed the full 20-day training protocol, demonstrating 100% adherence among this group.

### Study Design

This VR game was developed using the Unity (Unity Technologies) game engine and is compatible with the Oculus Quest 2 (Meta) VR device. The VR game app was designed for home-based cognitive control training, with each participant expected to engage in at least 20 minutes of training daily for 20 days. Research assistants monitored training data and proactively addressed difficulties over the phone each day, conducting in-home visits as needed for technical challenges.

This study included 3 assessment phases comprising a pretest for initial assessment, a posttest after 20 days of training, and a follow-up test conducted 3 months later. To evaluate the retest effect specifically, 14 participants underwent an additional test (retest) without training. Participants, including those in the no training retest group, were alternately allocated based on enrollment order with randomization. After evaluations, this group participated in training sessions, followed by subsequent posttests and follow-up assessments.

The pretest included the Korean Wechsler Intelligence Scale for Children-Fourth Edition (K-WISC-IV) [32], the Korean Color Trails test (CTT) 1 and 2 [33], the Korean Stroop test [34], the NIH toolbox [35], and the Korean Child Behavioral Checklist (K-CBCL) [36], lasting approximately 90 minutes excluding the parent-completed K-CBCL. The posttest and follow-up tests included CTT 1 and 2 [33], Stroop test, Flanker test from the NIH toolbox [35], and the K-CBCL [36], each taking about 30 minutes, excluding the parent-completed K-CBCL [36]. Training monitoring and assessments were conducted by research assistants who had graduated from master’s programs specializing in clinical psychology.

### Measurements

#### K-WISC-IV Assessment

The K-WISC-IV [32] uses 10 core subtests to measure intelligence. The subtests yield scaled scores (mean 10, SD 3) and 4 composite indices, such as verbal comprehension index (VCI), perceptual reasoning index (PRI), working memory index (WMI), and processing speed index (PSI), presented as standard scores (mean 100, SD 15).

#### Korean Version of the Color Trails Test 1 and 2

The Color Trails test [33] comprises 2 parts. The first (CTT1) part assesses attention sustainability, and the second (CTT2) part assesses the ability to shift attention. Converted T-scores based on the completion time for both assessment parts were used for analysis.

#### Korean Version of the Stroop Color-Word Test for Children

The Stroop Color-Word test [34] evaluates the ability to inhibit irrelevant information and select relevant responses. The Korean version, which demonstrated reliability (Cronbach α=0.72 for the normative population and 0.73 for the clinical population), was administered. Normative data are available for children aged 5-14 years. In this study, the number of correctly reported color words within 45 seconds served as the measurement.

#### NIH Toolbox Flanker Test

The Flanker Inhibitory Control test from the National Institutes of Health (NIH) toolbox [35] was used to evaluate executive function. This task assesses a participant’s ability to inhibit irrelevant visual information while maintaining attention on a target. The age-corrected standard scores (range 59‐140) were used for analysis.

#### K-CBCL Assessment

The K-CBCL [36] was used to identify behavioral problems in children, adapted from the Achenbach [37] Child Behavioral Checklist (CBCL). Raw scores were converted to T-scores (mean 50, SD 10). The analysis focused on the total problem score, attention problem score, and ADHD score.

#### Mobile-Based Cognitive Control Assessment App (CoCon)

CoCon [30] served to screen high-risk groups in the community. Its concurrent validity was established through comparisons with traditional neuropsychological tests, including the Stroop test and the CTT. Four composite indices were produced, converted into T-scores (mean 50, SD 10): sustained attention, working memory, cognitive control, and cognitive execution. A T-score of 40 was used as the cutoff for high-risk group selection (higher scores indicating better function).

### Statistical Analysis

Data analysis was performed using Jamovi software [38]. Demographic data were summarized by calculating means, SDs, and frequencies. Game-related behaviors were analyzed, including login frequency, instances of exiting games midway, total game time, total trial numbers, correct-incorrect counts, and reaction times. Paired 2-tailed *t* tests evaluated retest effects. The main analysis used repeated-measures ANOVAs to assess training effects at 3 time points, such as pretraining, posttraining, and 3 months posttraining. Scatter plots depicting pretest, posttest, and follow-up test results were generated using the *ggplot* function [39,40] in Jamovi software [38]. In addition, the Pearson correlation analysis was conducted between game-related behaviors and difference scores between pretests and follow-up tests in the Stroop test and CBCL total score, attentional problem score, and ADHD score. Training response patterns were analyzed using the *k*-means clustering method, applying Lloyd algorithm to identify distinct clusters based on standardized *z* scores (mean 0, SD 1) from difference scores between pretests and follow-up tests in the Stroop test and CBCL total score, attentional problem score, and ADHD score. Group comparisons were conducted using independent 2-tailed *t* tests across various parameters, including IQ subscales (verbal comprehension, perceptual reasoning, working memory, and processing speed) and game behaviors (correct/incorrect ratios, total response times, total trial numbers, number of exits during games, and login frequencies).

### Ethical Considerations

This study received approval from the institutional review board (SWU IRB-2020A-56; Multimedia Appendix 2). Written informed consent was obtained from participants and their parents, with each participant receiving 90,000 KRW (US $70) upon completing all evaluations. Data were anonymized.

## Results

### Participant Characteristics and Retest Effects

**Figure 2.**
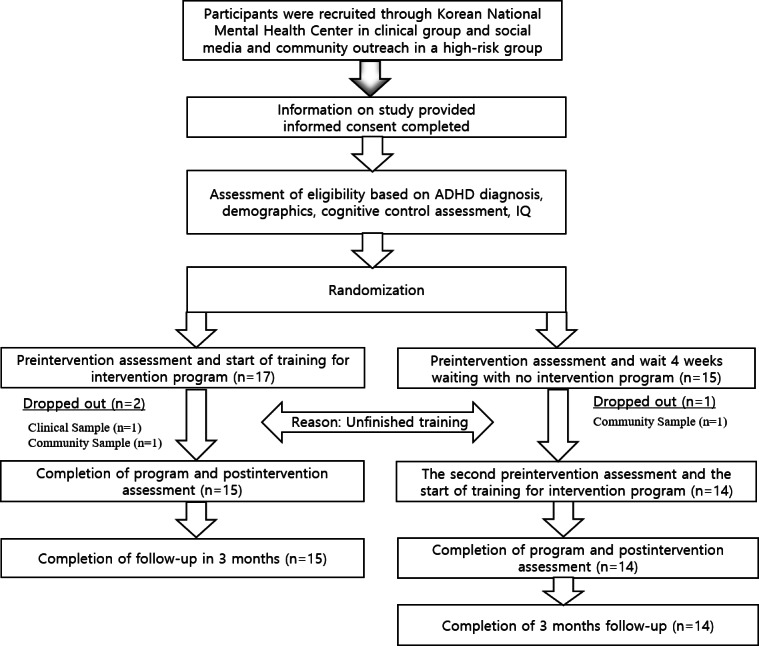
Flow diagram of the study. ADHD: attention-deficit/hyperactivity disorder.

Basic data analysis indicated that the mean age of the participants was 11.3 (SD 1.13) years, with ages ranging from 10 to 14 years (no retest group: 11.1, SD 1.28 years; no training retest group: mean 11.5, SD 0.94 years). A visual representation of the entire process depicting subsequent posttests and follow-up assessments can be found in Figure 2. The mean total IQ (K-WISC-IV) was 94.2 (SD 16.5; no retest group: mean 95.4, SD 15.97; no training retest group: 93, SD 17.62). The gender distribution among the 29 particpants comprised 21 (72%) boys and 8 (28%) girls. There was no significant group difference regarding gender ratio, with a chi-square (*χ*²) test resulting in *P*=.07.

The retest effects, assessed using paired *t* tests, revealed no significant changes between initial assessments and reevaluations without intervention across various measures (*t*_13_=−1.979, *P*=.07 for CTT 1; *t*_13_=−0.711, *P*=.49 for CTT 2; *t*_13_=−0.899, *P*=.39 for Flanker test from NIH toolbox; *t*_13_=−1.773, *P*=.10 for Stroop test; *t*_13_=1.224, *P*=.30 for CBCL Total score; *t*_13_=1.694, *P*=.11 for CBCL Attention Problems score; and *t*_13_=1.224, *P*=.24 for CBCL ADHD score).

### Effects of VR Training on Cognitive and Behavioral Measures

**Figure 3.**
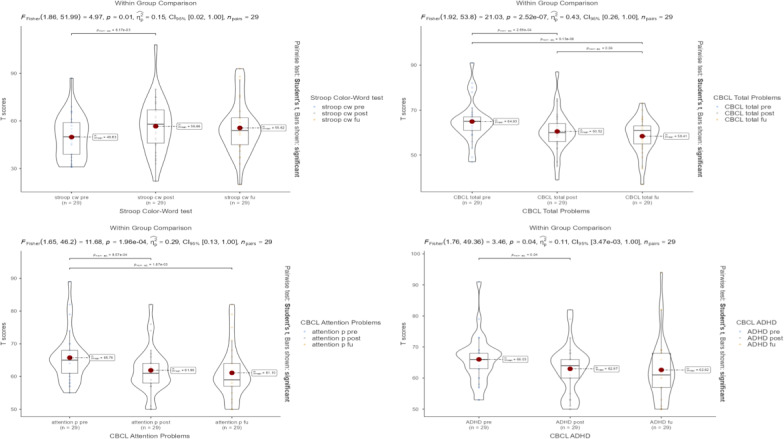
Scatterplots of Stroop test and CBCL subscale scores at pretest, posttest, and follow-up. ADHD: attention-deficit/hyperactivity disorder; CBCL: Child Behavior Checklist.

Table 1 details the observed training effects across pretest, posttest, and follow-up assessments. Repeated measures ANOVA revealed significant effects in the Stroop test (*F*_2,56_=4.97; *P*=.01; ηp^2^=0.151; pretest<posttest *P*=.006), CBCL Total Problems Score (*F*_2,56_=21.0; *P*<.001; ηp^2^=0.429; pretest<posttest *P*<.001; pretest<follow-up test *P*<.001), CBCL Attention Problems Score (*F*_2,56_=11.7; *P*<.001; ηp^2^=0.294; pretest<posttest *P*<.001; pretest<follow-up test *P*=.002), and CBCL ADHD score (*F*_2,56_=3.46; *P*=.04; ηp^2^=0.110; pretest<posttest *P*=.03). Figure 3 presents plots comparing the within-group results from the pretest, posttest, and follow-up tests, created using *ggplot2*. CTT1 and CTT2 and the Flanker test from the NIH toolbox did not demonstrate any significant effects among the pretest, posttest, and follow-up test.

**Table 1.** Repeated measures ANOVA results comparing pretest, posttest, and follow-up.

Measure and group		Pretest, mean (SD)	Posttest, mean (SD)	Follow-up, mean (SD)	*F* (*df*)	*P* value	ηp2
CTT^a^1					0.345 (2, 56)	.71	0.012
Total		54.6 (8.93)	55.9 (8.82)	55.9 (10.1)			
Group 1		57.3 (9.00)	58.8 (6.70)	58.8 (7.43)			
Group 2		51.7 (8.20)	56.3 (7.27)	52.9 (11.9)			
CTT2					1.29 (2, 56)	.28	0.044
Total		50.8 (7.29)	51.5 (8.82)	53.4 (8.86)			
Group 1		52.4 (7.02)	48.7 (11.7)	52.5 (7.86)			
Group 2		49.1 (7.43)	50.6 (10.5)	54.4 (10.0)			
Stroop Color-Word					4.97 (2, 56)	.01^b^	0.151
Total		49.8 (14.0)	56.7 (17.4)	55.6 (16.5)			
Group 1		52.6 (10.6)	56.9 (13.1)	54.6 (15.9)			
Group 2		46.9 (14.0)	56.4 (21.6)	56.7 (17.7)			
NIH^c^ Flanker test					1.76 (2, 56)	.18	0.059
Total		104 (17.5)	109 (19.0)	107 (18.2)			
Group 1		101 (16.3)	106 (15.4)	103 (16.5)			
Group 2		107 (18.9)	110 (19.6)	112 (19.5)			
CBCL^d^ Total Problems					21.0 (2, 56)	<.001^e^	0.429
Total		64.9 (9.26)	60.5 (9.23)	58.4 (7.94)			
Group 1		63.7 (9.43)	59.7 (11.2)	57.1 (8.76)			
Group 2		66.3 (9.22)	65.0 (9.47)	59.8 (7.01)			
CBCL Attentional Problems					11.7 (2, 56)	<.001^e^	0.294
Total		65.8 (7.61)	61.9 (7.10)	61.1 (7.99)			
Group 1		64.0 (5.59)	60.7 (7.45)	60.6 (7.70)			
Group 2		67.6 (9.15)	65.1 (8.48)	61.6 (7.70)			
CBCL ADHD^f^					3.46 (2, 56)	.03^b^	0.110
Total		66.0 (7.71)	63.0 (7.93)	62.6 (10.2)			
Group 1		64.3 (5.78)	61.5 (8.37)	61.8 (8.71)			
Group 2		67.9 (9.22)	66.6 (8.85)	63.5 (11.9)			

a CTT: Color Trails test.

b Statistically significant at *P*<.05.

c NIH: National Institutes of Health.

d CBCL: Child Behavior Checklist.

e Statistically significant at *P*<.001.

f ADHD: attention-deficit/hyperactivity disorder.

### Analysis of Training Response Patterns

**Figure 4.**
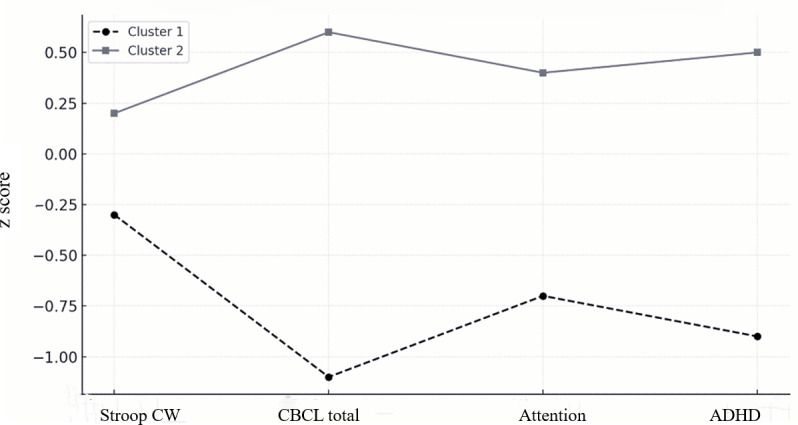
Clusters identified based on *z* scores of changes from pretest to follow-up. ADHD: attention-deficit/hyperactivity disorder; CBCL: Child Behavior Checklist; CW: color-word

A Pearson correlation analysis revealed significant positive correlations between the correct response ratio and the difference scores on the CBCL Total Problems, Attention Problems, and ADHD scales. Furthermore, significant correlations were found between the mean correct response time and the difference scores on the Stroop Color-Word test, as well as the CBCL Attention Problems and ADHD scales. The total login frequency was also significantly correlated with the difference scores on all 3 CBCL scales (see Table S1 in Multimedia Appendix 3 for details). The k-means cluster analysis, which used *z* scores of the difference between pretest and follow-up on the Stroop test and CBCL scales, produced 2 distinct clusters. Cluster 1 (n=10) did not show significant improvement, while cluster 2 (n=19) exhibited overall improvements from pretest to follow-up. A comparative analysis did not reveal any significant differences between the 2 clusters in age or on the IQ-related indices. However, there was a borderline significant difference in gender distribution between the clusters (*χ*²_1_=3.84; *P*=.05). Furthermore, a significant association was found between sample type (clinical vs community) and cluster membership (*χ*²_1_=8.03; *P*=.005) (see Tables S1 and S2 in Multimedia Appendix 4 for details). In addition, there were significant differences in game-related behavioral variables, including mean correct response time (*t*_27_=−2.56; *P*=.02) and the correct response ratio (*t*_27_=2.60; *P*=.02). These results suggest that a longer correct response time and a lower correct response ratio were associated with better training outcomes. The detailed results are presented in Table 2 and Figure 4.

**Table 2.** Cluster comparisons of IQ and game-related variables.

Variable (cluster)				Mean (SD)		*t* test (*df*)	*P* value
Age						1.89 (27)	.07
1				11.8 (1.32)			
2				11.0 (0.94)			
K-WISC-IV^a^							
Verbal comprehension						–0.636 (27)	.53
1				97.2 (24.93)			
2				102.2 (17.62)			
Perceptual reasoning						0.84 (27)	.41
1				104.4 (14.27)			
2				99.1 (16.61)			
Working memory						–1.18 (27)	.25
1				86.2 (21.56)			
2				94.1 (14.35)			
Processing speed						1.66 (27)	.11
1				92.4 (14.29)			
2				84.4 (11.06)			
Total IQ						–0.03 (27)	.97
1				94.1 (21.97)			
2				94.3 (13.55)			
Total trials						–1.10 (27)	.32
1				2617.3 (2576.5)			
2				3055.2 (2793.0)			
Game-related variables							
Game performance							
Total RT^b^						–1.14 (27)	.17
1				3319.4 (2914.1)			
2				5036.2 (3531.6)			
Correct RT (mean)						–2.56 (27)	.02^c^
1				1.2 (1.16)			
2				1.4 (1.33)			
Correct response ratio						2.60 (27)	.02^c^
1				77.3 (78.2)			
2				67.7 (70.8)			
Game behavior							
Login frequency						–1.98 (27)	.06
1				71.6 (61.5)			
2				117.7 (95.0)			
Frequency of midgame exit						0.32 (27)	.74
1				118.4 (108.0)			
2				112.8 (114.0)			
The RT of the first response						0.84 (27)	.41
1				0.8 (0.505)			
2				0.6 (0.460)			

a K-WISC-IV: Korean Wechsler Intelligence Scale for Children, Fourth Edition.

b RT: reaction time.

c Statistically significant at *P*<.05.

## Discussion

### Principal Findings

This pilot study provides preliminary evidence that a novel, at-home VR cognitive training game can significantly improve cognitive control and parent-reported ADHD symptoms in children. Notably, these therapeutic gains were sustained at a 3-month follow-up, and an analysis of a nonintervention group confirmed that the improvements were not solely attributable to test-retest effects. The study also demonstrated high feasibility. The integration of a telemonitoring system was effective in maintaining excellent adherence to the 20-day protocol, overcoming common challenges associated with remote interventions.

However, the Flanker test from the NIH toolbox and the CTT did not show significant training effects. This lack of significant findings may stem from the nature of these tasks, which primarily assess more automatic attentional processes, in contrast to the Stroop test, which targets controlled attention. Prior research has suggested that basic attention networks can exhibit unstable changes with repeated testing, making such measures potentially less sensitive to short-term training improvements [41]. A k-means clustering analysis was conducted based on changes observed in the Stroop Color-Word test, CBCL total behavioral scores, attentional problems, and ADHD symptom scores. This analysis revealed distinct patterns of training response among the identified clusters. Overall, the 2 clusters displayed contrasting trends: one group demonstrated a positive training effect, while the other showed no clear improvement. Notably, there were no significant differences in age and IQ indices between the clusters, suggesting that age and general intelligence did not account for the observed variability in training outcomes. Interestingly, the positively responding cluster exhibited longer correct response times and lower correct response ratios compared to the nonresponsive group. Although these findings appear counterintuitive, they may reflect greater engagement or more sincere participation, indicated by a higher number of total trials and longer reaction times, which could underlie the observed improvements. Notably, a significant association was found between the participant source (clinical vs community) and training response, with the community sample being less responsive to the training than the clinical sample. While the baseline assessment showed no significant differences between the 2 groups in IQ, age, or attention-related problems, the difference in training outcomes may suggest lower motivation in the community sample. However, given the small size of the community group, this interpretation is speculative and requires further investigation.

### Limitations

Despite these compelling findings, several critical limitations must be acknowledged. First, the study lacked a control group, which limits the ability to draw causal conclusions about the effects of the training. The within-subjects design, without a parallel control, restricts the interpretation of training efficacy in a more systematic and rigorous manner. Second, the relatively small sample size (N=29), along with imbalanced participant characteristics, particularly in terms of gender distribution and medication status, reduces the generalizability and statistical power of the findings. Third, the study did not assess higher-order cognitive functions, such as executive functioning. Incorporating such measures could have provided a more comprehensive understanding of the relationship between neuropsychological test performance and parent-reported behavioral outcomes.

### Conclusion

In conclusion, the newly developed VR-based cognitive control game, enhanced with a modified adaptive staircase algorithm and supported by a telemonitoring system, demonstrated its potential as an accessible and effective at-home intervention for children with ADHD symptoms. Building on research showing that user characteristics influence engagement with tele-mental health services [42], an important next step is to explore which specific user profiles benefit most from this VR-based intervention.

## Supplementary material

10.2196/66617 Multimedia Appendix 1 Comparisons between clinical and community participants.

10.2196/66617 Multimedia Appendix 2 Institutional review board approval.

10.2196/66617 Multimedia Appendix 3 Correlations between game behavior and difference scores of major measurements.

10.2196/66617 Multimedia Appendix 4 Contingency tables of cluster memberships.

## Abbreviations

ADHD: attention-deficit/hyperactivity disorder
CBCL: Child Behavior Checklist
CTT: Color Trails test
K-CBCL: Korean Child Behavior Checklist
K-WISC-IV: Korean Wechsler Intelligence Scale for Children, Fourth Edition
NIH: National Institutes of Health
PRI: perceptual reasoning index
PSI: processing speed index
PSI: processing speed index
VCI: verbal comprehension index
VR: virtual reality
WMI: working memory index
