# Supplementary material for: Effectiveness of Virtual Reality–Based Cognitive Control Training Game for Children With Attention-Deficit/Hyperactivity Disorder Symptoms: Preliminary Effectiveness Study
Source: JMIR Pediatr Parent. 2025 Sep 19;8:e66617. doi: 10.2196/66617 (PMC12448256; doi:10.2196/66617)
Supplement: Multimedia Appendix 1 [file pediatrics-v8-e66617-s001.docx]

Multimedia Appendix 1

|  | **Group ^a^** | **N** | **Mean** | **SD** | **t(27)** | **P** |
| --- | --- | --- | --- | --- | --- | --- |
| Age | 1 | 21 | 11.2 | 0.995 | -0.286 | 0.777 |
|  | 2 | 8 | 11.4 | 1.51 |  |  |
| K-WISC-IV Total Score | 1 | 21 | 94.3 | 15.164 | 0.047 | 0.962 |
|  | 2 | 8 | 94.0 | 20.88 |  |  |
| Vocabulary Comprehension Index Score | 1 | 21 | 103.4 | 18.710 | 1.275 | 0.213 |
|  | 2 | 8 | 92.9 | 23.02 |  |  |
| Perceptual Reasoning Index Score | 1 | 21 | 100.2 | 15.725 | -0.396 | 0.695 |
|  | 2 | 8 | 102.9 | 16.86 |  |  |
| Working Memory Index Score | 1 | 21 | 89.9 | 14.224 | -0.765 | 0.451 |
|  | 2 | 8 | 95.4 | 24.14 |  |  |
| Processing Speed Index Score | 1 | 21 | 85.8 | 12.526 | -0.974 | 0.338 |
|  | 2 | 8 | 90.9 | 12.92 |  |  |
| Stroop Test Color-Word Score(Pretest) | 1 | 21 | 48.3 | 12.92 | -0.959 | 0.346 |
|  | 2 | 8 | 53.9 | 16.76 |  |  |
| CBCL Total Problems Score (Pretest) | 1 | 21 | 64.6 | 8.812 | -0.333 | 0.741 |
|  | 2 | 8 | 65.9 | 10.93 |  |  |
| CBCL Attention Problems Score(Pretest) | 1 | 21 | 65.0 | 7.864 | -0.810 | 0.425 |
|  | 2 | 8 | 67.6 | 7.03 |  |  |
| CBCL ADHD Score (Pretest) | 1 | 21 | 66.6 | 7.846 | 0.600 | 0.553 |
|  | 2 | 8 | 64.6 | 7.65 |  |  |

Supplementary Table1. Comparisons between Clinical and Community participants

^a^ Group 1=Clinical Sample, Group 2=Community Sample
