# Supplementary material for: Effectiveness of Virtual Reality–Based Cognitive Control Training Game for Children With Attention-Deficit/Hyperactivity Disorder Symptoms: Preliminary Effectiveness Study
Source: JMIR Pediatr Parent. 2025 Sep 19;8:e66617. doi: 10.2196/66617 (PMC12448256; doi:10.2196/66617)
Supplement: Multimedia Appendix 2 [file pediatrics-v8-e66617-s002.pdf]

Notification of Research Plan Review Result

Recipient

Category	Name	Affiliation	Position	Field of Expertise
Principal Investigator	Hyunjoo Song	Seoul Women's University	Professor	Clinical Psychology

Sponsoring Agency: Ministry of Science and ICT

Submission Number: SWU IRB – 2020 - 73

IRB Approval Number: SWU IRB – 2020A - 56

Research Title

Development of a Mobile VR–Neuropsychological Paradigm for Early Identification of Cognitive Control Vulnerable Groups and Enhancement of Brain Function in Children and Adolescents

Research Summary

☑ Human Subject Research

☐ Human Biological Material Research

☐ Survey ☐ Interview ☐ Observational Research

☑ Interventional Study (Experimental vs. Control Group)

☐ Invasive Experimental Study (e.g., blood draw, medication, ingestion)

☐ Drawing Test ☐ Other ( )

☐ Blood ☐ Tissue ☐ Body Fluid ☐ Other ( )

- Collection Amount:

☐ Direct Collection ☐ Purchased from Vendor

☐ Supplied from Human Biological Materials Bank

☐ Provided by Other Researchers

☐ Other:

- Number of Research Subjects: Total of 60

Review Type

☐ Full Board Review

☐ Expedited Review

☑ Expedited (Simplified) Review

Review Date: February 25, 2021

Items Reviewed

☐ New Research Plan

☑ Revised Research Plan

☐ Change in Research Plan

☐ Continuing Research

☐ Report on (Early) Termination

☐ Serious Adverse Event Report

☐ Annual Continuing Review of Approved Research

☐ Protocol Deviation/Violation Report

☐ Provision of Human Biological Materials

☐ Disposal/Transfer of Human Biological Materials

☐ Other:

Review Result

☑ Approved

☐ Conditionally Approved

☐ Re-review Required

☐ Rejected

☐ Deferred

☐ Suspension or Termination of Previously Approved Research

☐ Other (   )

Approval Date: March 4, 2021

Approval Validity Period: Until December 31, 2021

Reporting Period

☐ Every 3 months

☐ Every 6 months

☑ Every year

☐ Other (   )

Review Comments

March 4, 2021

Chairperson, Institutional Review Board

Seoul Women's University

(Signature/Seal)
