# Supplementary material for: Effectiveness of Virtual Reality–Based Cognitive Control Training Game for Children With Attention-Deficit/Hyperactivity Disorder Symptoms: Preliminary Effectiveness Study
Source: JMIR Pediatr Parent. 2025 Sep 19;8:e66617. doi: 10.2196/66617 (PMC12448256; doi:10.2196/66617)
Supplement: Multimedia Appendix 3 [file pediatrics-v8-e66617-s003.docx]

| Table 1. Correlations between game behavior and difference scores of major measurements. | | | | | |
| --- | --- | --- | --- | --- | --- |
|  |  | Stroop Difference Score | CBCL Total Problems Difference Score | CBCL Attention Problems Difference Score | CBCL ADHD Difference Score |
| Correct Response Ratio | Pearson's r | -0.076 | **-0.426^a^** | **-0.396^a^** | **-0.477^b^** |
|  | df | 27 | 27 | 27 | 27 |
|  | P-value | 0.696 | 0.021 | 0.033 | 0.009 |
| Mean of Correct Response Time | Pearson's r | **0.400^a^** | 0.336 | **0.519^b^** | **0.589 ^c^** |
|  | df | 27 | 27 | 27 | 27 |
|  | P-value | 0.032 | 0.075 | 0.004 | <.001 |
| Login frequency | Pearson's r | -0.075 | **0.464^a^** | 0.33 | **0.381^a^** |
|  | df | 27 | 27 | 27 | 27 |
|  | P-value | 0.698 | 0.011 | 0.08 | 0.042 |
| Frequency of Mid-Game Exit | Pearson's r | 0.208 | -0.212 | -0.154 | -0.135 |
|  | df | 27 | 27 | 27 | 27 |
|  | P-value | 0.278 | 0.27 | 0.425 | 0.483 |
| *.* ^a^ P < .05, ^b^ P < .01, ^c^ P< .001 | | | | | |
