# Supplementary material for: Effectiveness of Virtual Reality–Based Cognitive Control Training Game for Children With Attention-Deficit/Hyperactivity Disorder Symptoms: Preliminary Effectiveness Study
Source: JMIR Pediatr Parent. 2025 Sep 19;8:e66617. doi: 10.2196/66617 (PMC12448256; doi:10.2196/66617)
Supplement: Multimedia Appendix 4 [file pediatrics-v8-e66617-s004.docx]

**Contingency Tables of Cluster Memberships**

**Table 1. Contingency Tables of Gender between Cluster Memberships**

|  | | Clusters Membership | |  |
| --- | --- | --- | --- | --- |
| Gender |  | Response | Non-Response | Total |
| Male | Observed | 16 | 5 | 21 |
|  | Expected | 13.76 | 7.24 | 21 |
| Female | Observed | 3 | 5 | 8 |
|  | Expected | 5.24 | 2.76 | 8 |
| Total | Observed | 19 | 10 | 29 |
|  | Expected | 19 | 10 | 29 |

**Table 2. Contingency Tables of between Sample Type (clinical vs. community) and Cluster Memberships**

| Sample Type |  | Cluster membership | |  |
| --- | --- | --- | --- | --- |
|  |  | Response | Non-Response | Total |
| Clinical Sample | Observed | 17 | 4 | 21 |
|  | Expected | 13.76 | 7.24 | 21 |
| Community Sample | Observed | 2 | 6 | 8 |
|  | Expected | 5.24 | 2.76 | 8 |
| Total | Observed | 19 | 10 | 29 |
|  | Expected | 19 | 10 | 29 |
